# *trans*-Di-μ-iodido-bis­[(3*H*-1,2-benzodithiole-3-thione)iodidomercury(II)]

**DOI:** 10.1107/S1600536809032152

**Published:** 2009-08-19

**Authors:** El Adoui Laifa, Lamia Bendjeddou, Naouel Boudraa, Slimane Dahaoui, Claude Lecomte

**Affiliations:** a Laboratoire de Chimie Moléculaire, du Contrôle, de l’Environnement et des Mesures Physico-Chimiques, Faculté des Sciences, Département de Chimie, Université Mentouri de Constantine, 25000 Constantine, Algeria; b Cristallographie, Résonance Magnétique et Modélisation (CRM2), Université Henri Poincaré, Nancy 1, Faculté des Sciences, BP 70239, 54506 Vandoeuvre lès Nancy CEDEX, France

## Abstract

The complete molecule of the dinuclear title compound, [Hg_2_I_4_(C_7_H_4_S_3_)_2_], is generated by crystallographic inversion symmetry. The complex has a dimeric structure in which each Hg^II^ ion adopts a tetra­hedral geometry and is coordinated by two bridging I atoms, one terminal iodide ion and one thio­carbonyl S atom (C=S) of the ligand. The square plane formed by the Hg and I atoms and their symmetry counterparts makes a dihedral angle of 89.66 (3)° with the DDT plane. There is no classical hydrogen bonding, but weak S⋯S inter­actions of 3.4452 (7) and 3.6859 (7) Å maintain the cohesion of the crystal structure.

## Related literature

For S⋯S inter­actions in sulfur-rich organic donor–acceptor compounds or radical salts, see: Cassoux *et al.* (1991 ▶); Klinsberg & Schraber (1962 ▶). For the effects on the mol­ecular packing and S⋯S contacts of modifying the structure of the organic molecule or changing the counter-ion or the co-crystallized solvent, see: Pullen & Olk (1999 ▶); Schlueter *et al.* (1996 ▶). In this context, a series of polymeric complexes has been reported with Ag^+ ^(Dai, Munakata, Kuroda-Sowa *et al.*, 1997 ▶; Dai, Kudora-Sowa *et al.*, 1997 ▶) and a tetra-nuclear CuI_4_ cluster (Dai, Munakata, Wu *et al.*, 1997 ▶). For comparison bond lengths and angles in related chloride-bridged dimeric Hg(II) complexes, see: Brodersen & Hummel (1987 ▶); Dean (1978 ▶).
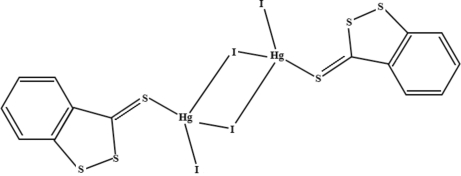


## Experimental

### 

#### Crystal data

[Hg_2_I_4_(C_7_H_4_S_3_)_2_]
*M* *_r_* = 1277.40
Triclinic, 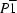

*a* = 7.86285 (10) Å
*b* = 8.19304 (11) Å
*c* = 10.46506 (13) Å
α = 105.2917 (11)°
β = 98.3031 (10)°
γ = 105.6957 (11)°
*V* = 608.92 (2) Å^3^
*Z* = 1
Mo *K*α radiation
μ = 18.18 mm^−1^
*T* = 100 K
0.16 × 0.1 × 0.08 mm

#### Data collection

Oxford Diffraction SuperNova diffractometer with an Atlas detector
Absorption correction: analytical (Clark *et al.*, 1995 ▶) *T*_min_ = 0.167, *T*_max_ = 0.347
44929 measured reflections
4951 independent reflections
4698 reflections with *I* > 2σ(*I*)
*R*_int_ = 0.051

#### Refinement

*R*[*F* ^2^ > 2σ(*F* ^2^)] = 0.015
*wR*(*F* ^2^) = 0.037
*S* = 1.05
4951 reflections
118 parameters
H-atom parameters constrained
Δρ_max_ = 1.20 e Å^−3^
Δρ_min_ = −1.55 e Å^−3^

### 

Data collection: *CrysAlis CCD* (Oxford Diffraction, 2009 ▶); cell refinement: *CrysAlis RED* (Oxford Diffraction, 2009 ▶); data reduction: *CrysAlis RED*; program(s) used to solve structure: *SIR92* (Altomare *et al.*, 1993 ▶); program(s) used to refine structure: *SHELXL97* (Sheldrick, 2008 ▶); molecular graphics: *ORTEP*-3I (Farrugia, 1997 ▶); software used to prepare material for publication: *WinGX* (Farrugia, 1999 ▶).

## Supplementary Material

Crystal structure: contains datablocks global, I. DOI: 10.1107/S1600536809032152/bq2156sup1.cif

Structure factors: contains datablocks I. DOI: 10.1107/S1600536809032152/bq2156Isup2.hkl

Additional supplementary materials:  crystallographic information; 3D view; checkCIF report

## Figures and Tables

**Table:** 

Hg1—S1	2.5169 (7)
Hg1—I2	2.6599 (2)
Hg1—I1	2.8148 (4)
Hg1—I1^i^	3.1002 (4)

**Table:** 

S1—Hg1—I2	138.842 (15)
S1—Hg1—I1	98.374 (17)
I2—Hg1—I1^i^	103.851 (13)
I1—Hg1—I1^i^	95.210 (10)

Symmetry code: (i) 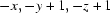.

**Fig. 1.**
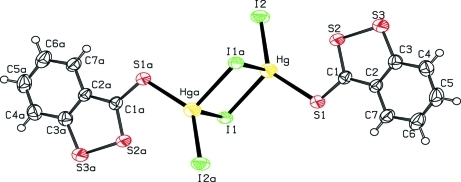
: An ORTEP view of the dimeric structure of (I) with the atom-labeling scheme. Displacement ellipsoids for non-H atoms are drawn at the 50% probability level. [Symmetry code: (a) -x, 1-y, 1-z]

**Fig. 2.**
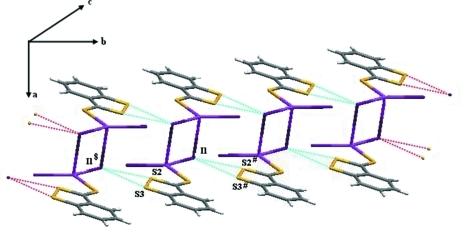
: A View of the structure compound showing the interactions between two adjacent dimer. Atoms marked with a hash symbol (#) and dollar sign ($), are at the symmetry positions (x, 1 + y, z), (x, -1 + y, z) respectively.

## supplementary crystallographic information

### Comment

Sulfur-rich organic donor-acceptor compounds or radical salts are widely used as bricks in the crystal architecture of molecular materials. The S···S interaction or contact in this compounds is one of the most important contributors to their unique electronic properties (Klinsberg *et al.*, 1962, Cassoux *et al.*, 1991). It has been understood that intermolecular S···S interactions are providing the pass way of the electrons in the molecular conductor. Up to now great efforts have been made to design the molecular packing and to strengthen the S···S contacts by modifying the structure of the organic molecule itself, by changing the size of the conter ion and even the co-crystallized solvent molecules (Pullen *et al.*, 1999; Schlueter *et al.*, 1996). In this context a series of polymeric complexes have been reported with (Ag+) metal (Dai, Munakata, Kuroda-Sowa *et al.*, 1997; Dai, Kudora-Sowa *et al.*, 1997) and a tetra-nuclear cluster CuI4 (Dai, Munakata, Wu *et al.*, 1997). In this paper an organic-inorganic hybrid compound is reported with the formula: [Hg_2_I_4_(DTT)_2_](1),DTT=C_7_H_4_S_3_, 4,5-benzo-1,2-dithiole-3-thione.

The complex [Hg_2_ (C_14_H_8_S_6_)I_4_] is found to be a halogen-bridged dimer related by an inversion centre. An *ORTEP* view of the complex together with the atomic labeling scheme is given in Fig.1. The mercury atom is four-coordinated with significant distortion from tetrahedral. Two of these bonds are formed by two asymmetric iodide bridges, while the remaining two bonds are formed by a terminal iodide ion and thiocarbonyl sulfur atom of the ligand. The square-like plane formed by mercury and iodide atoms with their symmetry counterparts makes 89.66 (3)° dihedral angle with DDT plane.

The selected bond lengths and angles for the complex are listed in table (1) in which the data of the dimeric unit are comparable to those reported in the literature for related chloride bridged dimeric Hg(II) complexes (Brodersen *et al.*, 1987; Dean, 1978).

Coordination modes of the C_7_H_4_S_3_ ligand have been classified into four types (Dai, Kudora-Sowa *et al.*, 1997). They are monodentate coordination by the thiocarbonyl group (type I), bridge formation by the sulfur of the thiocarbonyl group (type II), bidentate coordination by both thiocarbonyl group and thioether group (type III) and tridentate coordination by the thiocarbonyl sulfur (acting as a bridge) and the thioether sulfur (type IV).

Although the coordination of the ligand in type I has been found in complex: [Cu_4_I_4_(C_5_H_4_S_5_)_4_]∞ and{[Ag(C_5_H_4_S_5_)_3_]ClO_4_CH_3_CN}_2_ (Dai, Kudora-Sowa *et al.*, 1997; Dai, Munakata, Wu *et al.*, 1997), other coordination types co-exist in these complexes.

[Hg_2_(C_7_H_4_S_3_)_2_I_4_] is the complex found in the coordination of the ligands only in type I. The S(2)—C(1)distance of 1.703 (2)Å in this compound is longer than that for the free ligand 1.645Å (Dai, Munakata, Kuroda-Sowa *et al.*, 1997). This longer S(2)—C(1) distance attributes to the strong coordination of the thiocarbonyl sulfur to soft mercury(II) ion that weakens the S(2)—C(1) bond.

The absence of intermolecular hydrogen bonding shows that the molecules are retained to each other by Van der Walls interaction only. The second type of bonds arises from an S(2)—I(1) and S(3)—I(1) interaction with two different bond lengths, 3.4452 (7)Å and 3.6859 (7) Å respectively (Fig.2). These bonds maintain the cohesion of the crystalline structure.

### Experimental

The reagent C_7_H_4_S_3_ was prepared using a literature method (Klinsberg *et al.*, 1962) and characterized. The solvent was dried and distilled by a standard method before use. All other chemicals were obtained from commercial sources and used without further purification. Infrared spectra were measured as KBr disc on a Nicolet 205 F T—IR spectrometer.

A solution of HgI_2_ (2.5 m mol, 1,130 g) in acetone (15 ml) was added to a solution of C_7_H_4_S_3 _(2.5 m mol, 0,460 g) in acetone at room temperature under argon atmosphere. An orange precipitate was formed immediately and the mixture was stirred for 50 min. The precipitate was filtered, washed with petroleum ether (yield: 60%). Orange crystals for x-ray measurement were obtained by recrystaling the solid in THF. IR (KBr, cm^-1^):n(C=C) 1447 ms, n(C=S) 991,3vs, n(Hg—S) 256,5 ms, n(Hg—I) 167,8vs.

### Refinement

H atoms were positioned geometrically and refined in the riding-model approximation, with C—H = 0.93 Å and with *U*_iso_(H) = 1.2 U_eq_(C)

### Crystal data

**Table:**

[Hg_2_I_4_(C_7_H_4_S_3_)_2_]	*Z* = 1
*M**_r_* = 1277.40	*F*(000) = 560
Triclinic, *P*1	*D*_x_ = 3.483 Mg m^−^^3^
Hall symbol: -P 1	Melting point: 192 K
*a* = 7.86285 (10) Å	Mo *K*α radiation, λ = 0.7107 Å
*b* = 8.19304 (11) Å	Cell parameters from 4951 reflections
*c* = 10.46506 (13) Å	θ = 2.8–35.0°
α = 105.2917 (11)°	µ = 18.18 mm^−^^1^
β = 98.3031 (10)°	*T* = 100 K
γ = 105.6957 (11)°	Needle, orange
*V* = 608.92 (2) Å^3^	0.16 × 0.1 × 0.08 mm

### Data collection

**Table:**

Super Nova diffractometer (Dual, Cu at zero, Mo active) with an Atlas detector	4951 independent reflections
Radiation source: Enhance (Mo) X-ray Source	4698 reflections with *I* > 2σ(*I*)
graphite	*R*_int_ = 0.051
Detector resolution: 10.4508 pixels mm^-1^	θ_max_ = 34.0°, θ_min_ = 3.2°
ω scans	*h* = −12→12
Absorption correction: analytical (Clark *et al.*, 1995)	*k* = −13→13
*T*_min_ = 0.167, *T*_max_ = 0.347	*l* = −16→16
44929 measured reflections	

### Refinement

**Table:**

Refinement on *F*^2^	0 restraints
Least-squares matrix: full	H-atom parameters constrained
*R*[*F*^2^ > 2σ(*F*^2^)] = 0.015	*w* = 1/[σ^2^(*F*_o_^2^) + (0.0169*P*)^2^ + 0.3949*P*] where *P* = (*F*_o_^2^ + 2*F*_c_^2^)/3
*wR*(*F*^2^) = 0.037	(Δ/σ)_max_ = 0.002
*S* = 1.05	Δρ_max_ = 1.20 e Å^−^^3^
4951 reflections	Δρ_min_ = −1.54 e Å^−^^3^
118 parameters	

### Special details

**Table:**

Geometry. All e.s.d.'s (except the e.s.d. in the dihedral angle between two l.s. planes) are estimated using the full covariance matrix. The cell e.s.d.'s are taken into account individually in the estimation of e.s.d.'s in distances, angles and torsion angles; correlations between e.s.d.'s in cell parameters are only used when they are defined by crystal symmetry. An approximate (isotropic) treatment of cell e.s.d.'s is used for estimating e.s.d.'s involving l.s. planes.
Refinement. Refinement of *F*^2^ against ALL reflections. The weighted *R*-factor *wR* and goodness of fit *S* are based on *F*^2^, conventional *R*-factors *R* are based on *F*, with *F* set to zero for negative *F*^2^. The threshold expression of *F*^2^ > σ(*F*^2^) is used only for calculating *R*-factors(gt) *etc*. and is not relevant to the choice of reflections for refinement. *R*-factors based on *F*^2^ are statistically about twice as large as those based on *F*, and *R*- factors based on ALL data will be even larger.

### Fractional atomic coordinates and isotropic or equivalent isotropic displacement parameters (Å^2^)

**Table:**

	*x*	*y*	*z*	*U*_iso_*/*U*_eq_	
Hg1	0.176661 (11)	0.378520 (10)	0.439759 (7)	0.01756 (2)	
I1	0.127512 (16)	0.692780 (15)	0.404010 (12)	0.01240 (3)	
I2	0.072031 (19)	0.088861 (17)	0.221273 (13)	0.01654 (3)	
S1	0.41650 (7)	0.51906 (6)	0.65918 (5)	0.01343 (8)	
C1	0.4481 (3)	0.3455 (3)	0.70715 (19)	0.0124 (3)	
S2	0.33319 (7)	0.13144 (6)	0.60634 (5)	0.01541 (9)	
S3	0.44554 (7)	0.00480 (7)	0.72353 (5)	0.01732 (9)	
C7	0.6773 (3)	0.5278 (3)	0.92676 (19)	0.0145 (3)	
H7	0.6733	0.6349	0.9138	0.017*	
C2	0.5686 (3)	0.3630 (3)	0.82988 (19)	0.0117 (3)	
C3	0.5762 (3)	0.2021 (3)	0.85055 (19)	0.0142 (3)	
C5	0.7939 (3)	0.3662 (3)	1.0612 (2)	0.0185 (4)	
H5	0.8692	0.369	1.1394	0.022*	
C6	0.7895 (3)	0.5283 (3)	1.0409 (2)	0.0173 (4)	
H6	0.8629	0.6363	1.1049	0.021*	
C4	0.6894 (3)	0.2039 (3)	0.9680 (2)	0.0176 (4)	
H4	0.6937	0.0976	0.9825	0.021*	

### Atomic displacement parameters (Å^2^)

**Table:**

	*U*^11^	*U*^22^	*U*^33^	*U*^12^	*U*^13^	*U*^23^
Hg1	0.02112 (4)	0.01604 (4)	0.01403 (4)	0.00738 (3)	−0.00021 (3)	0.00324 (3)
I1	0.01305 (5)	0.01087 (5)	0.01336 (5)	0.00370 (4)	0.00296 (4)	0.00420 (4)
I2	0.01919 (6)	0.01330 (5)	0.01626 (6)	0.00601 (5)	0.00372 (4)	0.00271 (4)
S1	0.0141 (2)	0.01235 (18)	0.01343 (19)	0.00436 (16)	0.00122 (15)	0.00450 (15)
C1	0.0114 (7)	0.0132 (7)	0.0130 (7)	0.0045 (6)	0.0037 (6)	0.0038 (6)
S2	0.0157 (2)	0.01270 (18)	0.0152 (2)	0.00376 (17)	−0.00177 (16)	0.00373 (16)
S3	0.0200 (2)	0.01284 (19)	0.0179 (2)	0.00602 (18)	−0.00073 (17)	0.00482 (17)
C7	0.0142 (8)	0.0160 (8)	0.0127 (8)	0.0052 (7)	0.0022 (6)	0.0037 (7)
C2	0.0104 (7)	0.0145 (7)	0.0110 (7)	0.0053 (6)	0.0026 (6)	0.0039 (6)
C3	0.0155 (8)	0.0166 (8)	0.0123 (7)	0.0076 (7)	0.0038 (6)	0.0050 (6)
C5	0.0187 (9)	0.0244 (9)	0.0135 (8)	0.0110 (8)	0.0014 (7)	0.0049 (7)
C6	0.0155 (9)	0.0198 (9)	0.0138 (8)	0.0054 (7)	0.0007 (7)	0.0026 (7)
C4	0.0200 (9)	0.0201 (9)	0.0143 (8)	0.0098 (8)	0.0025 (7)	0.0055 (7)

### Geometric parameters (Å, °)

**Table:**

Hg1—S1	2.5169 (7)	C7—C6	1.377 (3)
Hg1—I2	2.6599 (2)	C7—C2	1.409 (3)
Hg1—I1	2.8148 (4)	C7—H7	0.93
Hg1—I1^i^	3.1002 (4)	C2—C3	1.406 (3)
I1—Hg1^i^	3.1002 (4)	C3—C4	1.404 (3)
S1—C1	1.6935 (19)	C5—C4	1.373 (3)
C1—C2	1.430 (3)	C5—C6	1.408 (3)
C1—S2	1.702 (2)	C5—H5	0.93
S2—S3	2.0532 (7)	C6—H6	0.93
S3—C3	1.734 (2)	C4—H4	0.93
			
S1—Hg1—I2	138.842 (15)	C3—C2—C7	119.89 (17)
S1—Hg1—I1	98.374 (17)	C3—C2—C1	115.91 (17)
I2—Hg1—I1	117.574 (11)	C7—C2—C1	124.19 (17)
S1—Hg1—I1^i^	91.326 (17)	C4—C3—C2	120.60 (18)
I2—Hg1—I1^i^	103.851 (13)	C4—C3—S3	122.18 (16)
I1—Hg1—I1^i^	95.210 (10)	C2—C3—S3	117.21 (14)
Hg1—I1—Hg1^i^	84.790 (10)	C4—C5—C6	121.54 (18)
C1—S1—Hg1	105.26 (7)	C4—C5—H5	119.2
C2—C1—S1	124.72 (15)	C6—C5—H5	119.2
C2—C1—S2	115.08 (14)	C7—C6—C5	120.41 (19)
S1—C1—S2	120.20 (11)	C7—C6—H6	119.8
C1—S2—S3	97.66 (7)	C5—C6—H6	119.8
C3—S3—S2	94.06 (7)	C5—C4—C3	118.47 (19)
C6—C7—C2	119.08 (18)	C5—C4—H4	120.8
C6—C7—H7	120.5	C3—C4—H4	120.8
C2—C7—H7	120.5		

Symmetry codes: (i) −*x*, −*y*+1, −*z*+1.
